# Radiofrequency Ablation of Hepatocellular Carcinoma: A Literature Review

**DOI:** 10.4061/2011/104685

**Published:** 2011-05-11

**Authors:** Yasunori Minami, Masatoshi Kudo

**Affiliations:** Department of Gastroenterology and Hepatology, School of Medicine, Kinki University, 377-2 Ohno-Higashi, Osaka-Sayama 589-8511, Japan

## Abstract

Radiofrequency ablation (RFA) of liver cancers can be performed safely using percutaneous, laparoscopic, or open surgical techniques, and much of the impetus for the use of RFA has come from cohort series that have provided an evidence base for this technique. Here, we give an overview of the current status of radiofrequency ablation (RFA) for hepatocellular carcinoma (HCC), including its physical properties, to assess the characteristics that make this technique applicable in clinical practice. We review the technical development of probe design and summarize current indications and outcomes of reported clinical use. An accurate evaluation of treatment response is very important to secure successful RFA therapy since a sufficient safety margin (at least 0.5 cm) can prevent local tumor recurrences. We also provide a profile of side effects and information on the integration of this technique into the general management of patients with HCC. To minimize complications of RFA, physicians should be familiar with each feature of complication. Appropriate management of complications is essential for successful RFA treatment. Moreover, adjuvant therapy, such as molecular targeted therapies following curative therapy, is expected to further improve survival after RFA.

## 1. Introduction

Hepatic resection forms part of the conventional treatment for patients with hepatocellular carcinoma (HCC); however, the majority of primary liver cancers are not suitable for curative resection at the time of diagnosis. Difficulties of surgical resection may be related to size, site, and number of tumors, vascular and extrahepatic involvement as well as liver function of the patient [1–4]. There is a need to develop a simple and effective technique for the treatment of unresectable tumors within the liver. Therefore, local ablative techniques (percutaneous ethanol injection (PEI), microwave coagulation therapy (MCT), and radiofrequency ablation (RFA)) have emerged in clinical practice to expand the pool of patients considered for liver-directed therapies [5–8]. Especially, RFA is not associated with some of the side effects of other ablative techniques [9]. Thus, RFA is currently performed widely due to the ease of use, safety, reasonable cost, and applicability to minimally invasive techniques [10]. 

This paper reviews the evidence supporting the use of RFA for HCC.

## 2. Background

### 2.1. Localized Application of Radiofrequency Energy

RFA is a localized thermal treatment technique designed to induce tumor destruction by heating the tumor tissue to temperatures that exceed 60°C [11]. The alternating current of radiofrequency waves passing down from an uninsulated electrode tip into the surrounding tissues generates changes in the direction of ions and creates ionic agitation and frictional heating. This tissue heating then drives extracellular and intracellular water out of the tissue, resulting in tissue destruction by coagulative necrosis [12, 13]. When tumor cells are heated above 45–50°C, intracellular proteins are denatured and cell membranes are destroyed through dissolution and melting of lipid bilayers. As a result, successful ablations usually increase the temperature of the ablated tissue to above 60°C.

Percutaneous RFA under local anesthesia was feasible, although intraoperative RFA under general anesthesia was also performed to prevent severe pain and discomfort during the procedure.

### 2.2. RFA Electrodes and Generators

Three types of RF electrodes are currently available commercially: two brands of retractable needle electrodes (model 70 and model 90 Starburst XL needles, RITA Medical Systems, Mountain View, CA; LeVeen needle electrode, Boston Scientific, Boston, MA) and an internally cooled electrode (Cool-Tip RF electrode; Radionics, Burlington, MA) [14]. 

The needle electrodes of RITA consist of a 14-gauge insulated outer needle that houses nine retractable curved electrodes of various lengths. When the electrodes are extended, the device assumes the approximate configuration of a Christmas tree. Nine of the electrodes are hollow and contain thermocouples in their tips in order to measure the temperature of adjacent tissue. The alternating electric current generator comes in a 250 W model at 460 kHz (Model 1500X RF Generator, RITA Medical Systems). The ablation algorithm is based on the temperature at the tips of the electrodes. After the ablation cycle is completed, a temperature reading from the extended electrodes in excess of 50°C at 1 min is considered to indicate satisfactory ablation. 

Another RFA device (LeVeen Needle Electrode; Radiotherapeutics) has retractable curved electrodes and an insulated 17-gauge outer needle that houses 10 solid retractable curved electrodes that, when deployed, assume the configuration of an umbrella. The electrodes are manufactured in different lengths (2 to 4.0 cm umbrella diameter). The alternating electric current generator is 200 W operated at 480 kHz (RF 3000; Boston Scientific). The ablation algorithm is based on tissue impedance, and ablation is considered successful if the device impedes out. 

The third RFA device (Cool-Tip radiofrequency electrode; Radionics) has an insulated hollow 17-gauge needle with an exposed needle tip of variable length (2 or 3 cm). The tip of the needle contains a thermocouple to record the temperature of adjacent tissue. The shaft of the needle has two internal channels that allow the needle to be perfused with chilled water. In an attempt to further increase the size of the ablation area, the manufacturer placed three of the cooled needles in a parallel triangular cluster with a common hub. The generator has a peak power output of 200 W and is operated at 480 kHz (CC-1; Radionics). The ablation algorithm is based on tissue impedance, and ablation is considered successful if the device impedes out. As a result, successful ablations usually increase the temperature of the ablated tissue to above 60°C.

### 2.3. Treatment Algorithm in Japan and the West

RFA is basically recommended for HCC nodules with a maximum diameter of 3 cm in patients with not more than three tumors who are contraindicated for surgery, although the typical treatment algorithms in Japan, North America, and Europe are each slightly different [35].

One of the major treatment algorithms in Japan is the “consensus-based clinical practice manual for HCC” [14, 36] edited by the Japan Society of Hepatology (JSH). This consensus recommends (1) hepatectomy for a single tumor regardless of tumor size, but local treatment may be selected for a tumor 2 cm or smaller in Child-Pugh B patients; (2) hepatectomy or local treatment when there are 2 or 3 tumors and the tumor size is within 3 cm; (3) liver transplantation for Child-Pugh C patients with 3 or fewer tumors 3 cm or smaller or a single tumor with a tumor size within 5 cm (Milan Criteria); (4) RFA combined with transcatheter arterial chemoembolization (TACE) is recommended for tumors more than 3 cm in diameter. RFA is also recommended for 4 or more nodules where applicable. 

In Europe and North America, the algorithm established by the American Association of the Study of the Liver Disease (AASLD) [37] recommends local treatment for 3 or fewer 3 cm or smaller early-stage HCCs and 2-cm or smaller very-early-stage HCCs with complications, such as portal hypertension.

### 2.4. Assessment of Technical Effectiveness

The assessment of the therapeutic effect of RFA is very important. The technical effectiveness of ablation is commonly assessed by findings on contrast-enhanced CT or MRI. A tumor was considered to have been successfully ablated when there were no longer any enhanced regions within the entire tumor during the arterial phase and at least a 0.5 cm margin of apparently normal hepatic tissue surrounding the tumor during the portal phase [38–40]. Failure to establish a sufficient ablative safety margin was shown to be an independently significant risk factor for local tumor progression on multivariate analysis [41]. Part of the tumor was diagnosed as remaining viable when images of the ablated area showed nodular peripheral enhancement [42]. 

Basically, the local recurrence rate following a single RFA treatment depends on how strictly the therapeutic effect is assessed. In cases of HCC in which local curative therapy was achieved by securing a safety margin, the 4-year survival rate was relatively high, at 66%–82% (results in Japan) [35, 43].

## 3. Clinical Outcomes

### 3.1. Percutaneous Approach

#### 3.1.1. Survival: Comparison with Those after Resection

A randomized control trial (RCT) has shown that RFA achieved survival rates similar to those achieved by resection (Table 1) [15]. Chen et al. conducted RCT on 180 patients with a solitary HCC ≤5 cm indicated to receive either percutaneous RFA or surgical resection [15]. This study showed that percutaneous RFA achieved the same overall and disease-free survival rates as surgical resection for patients with small solitary HCC. The 1- and 4-year overall survival rates after percutaneous RFA and surgery were 95.8%, 67.9% and 93.3%, 64.0%, respectively. The corresponding disease-free survival rates were 85.9%, 46.4% and 86.6%, 51.6%, respectively. Recently, Huang et al. reported an RCT trial in which the 1-, 3-, and 5-year overall survival rates for the RFA group and the RES group were 86.96%, 69.57%, 54.78% and 98.26%, 92.17%, 75.65%, respectively. Overall survival and recurrence-free survival were significantly higher in the surgical resection group than in the RFA group (*P* = .001, *P* = .017). However, percutaneous RFA can be expected to have an advantage over liver resection in providing a better short-term postoperative result because local ablative therapy is a less invasive procedure [16–25].

#### 3.1.2. Local Controllability (Local Tumor Progression)

The local recurrence rate after RFA for HCC ranged from 1.7% to 41% [26–34] (Table 2). Local tumor progression is related to incomplete tumor ablation. It is often difficult to obtain a specific safety margin in three dimensions all around a large tumor. Some researchers reported that the most important factor associated with failure of local tumor control could be tumor size [8, 36–38]. In Table 2, local tumor progression did not necessarily depend on the tumor size; however, recurrence could occur even after a sufficient margin had been ensured. It is considered that local recurrence appears to arise from residual cancer after RFA while recurrence from a microsatellite or by microvascular invasion other than the main nodule may also appear as a late local recurrence. The local tumor progression rate can differ markedly depending on whether or not a 5 mm circumferential safety margin has been secured. Nishijima et al. categorized the presence of no margin, a partially lacking margin, margin narrower than 5 mm, and complete margin wider than 5 mm as R0, R1, R2, and R3 on the assessment of the therapeutic effect of RFA, respectively, and found significant differences in the local recurrence rate between R0 and R1 and between R2 and R3. The local recurrence rate significantly differed between patients with and without a sufficient safety margin [44]. Therefore, ensuring a safety margin in RFA is important for not only the simultaneous treatment of microsatellite lesions, but also to ensure sufficient tumor ablation on the assumption of a partial volume effect-associated limitation on evaluation of the therapeutic effect by imaging.

#### 3.1.3. Advances of Techniques: Large HCC

Tumor size is an important factor influencing the local recurrence rate after RFA [45]. To increase the size of the coagulation zone in RFA, physicians have tried using vascular occlusion during RFA because vascular occlusion reduces heat dispersion. It was shown in the consensus meeting “HCC Treatment” at the 45th Annual Meeting of the JSH in Kobe in 2009 [46] that about 90% of physicians performing RFA employ lipiodol TACE-preceded RFA for 3 cm or larger HCCs. Lipiodol TACE-preceded RFA is relatively curative and can be readily performed for the following reasons: (1) lipiodol regurgitates into the portal branches via the peribiliary venous plexus, causing a transient state of liver infarction, which reduces the cooling effect, expanding the ablative area, and resulting in (2) coagulation of satellite lesions [43]. Peng et al. reported a series of 120 patients with HCC, and the 1-, 3-, 5-year overall survival rates for TACE-preceded RFA and RFA groups were 93%, 75%, 50%, and 89%, 64%, 42%, respectively (*P* = .045) [47]. Yamakado et al. reported that the survival rates of large HCC cases treated with resection and lipiodol TACE-preceded RFA were almost equivalent [48]. TACE combined with RFA therapy might improve the overall survival status for patients with large HCCs (Table 3) [47, 49–52].

#### 3.1.4. Advanced Techniques: Tumors Abutting the Diaphragm and Gastrointestinal Tract

Ultrasound- (US-) guided procedures are necessary but limited for tumors located under the diaphragm. However, saline solution injection into the pleural cavity can separate the lung and liver on B-mode US, that is, artificial pleural effusion acts as an acoustic window. There are reports on the feasibility and safety of RFA with artificially induced pleural effusion for HCC located in the right subphrenic region [53–56]. In a series of 24 patients with HCC located in the hepatic dome, 200–1100 mL of 5% glucose solution was infused intrathoracically to separate the lung and liver, thus, complete tumor necrosis in a single session was achieved in 96.4% [56]. 

Artificial preparation of a space between the intestine and nodule by infusing normal saline or 5% glucose (artificial ascites method) for treatment has recently become possible [57, 58]. These techniques markedly expanded the indication for RFA. Laparoscopic resection or laparotomic RFA had to be inevitably performed in patients with HCC nodules <2.0 cm in diameter before the introduction of artificial ascites, but more than 90% of cases are now treatable by the “artificial ascites method”.

#### 3.1.5. Advanced Techniques: Cases That Are Unclear on B-Mode US

Multiple RFA sessions for HCCs were frequently required because of HCC nodules that are unclear on B-mode US. Under CT fluoroscopy using either CT arteriography or iodized oil injection, we can target and puncture hepatic malignancies using a percutaneous ethanol injection needle. Real-time CT fluoroscopy is useful to guide the needle puncture and to monitor ethanol injection in small hepatic malignancies [67]. Another merit is that the efficacy of treatment can be evaluated using contrast enhanced CT immediately after treatment. 

Contrast enhanced harmonic US imaging is able to evaluate small hypervascular HCCs even when B-mode US cannot adequately characterize the tumors [68–72]. The microbubbles of these contrast agents provide stable nonlinear oscillation in a low-power acoustic field because of the hard shells of these bubbles, producing great detail in the harmonic signals in real time [71–73]. It has been reported that contrast harmonic sonography-guided RFA is an efficient approach for guiding further ablation of hepatic malignancies that are not clearly demarcated by B-mode US [74–78]. 

Virtual CT sonography using magnetic navigation (Real-time Virtual Sonography (RVS); HITACHI Medico, Tokyo, Japan) provides cross sectional images of CT volume data corresponding to the angle of the transducer in the magnetic field in real-time. This imaging technique displays a real-time synchronized multiplanar CT image in precisely the same slice of the US plane. Thus, RVS can be used for real-time needle insertion guidance, especially for nodules demonstrated on CT, but not on US [79, 80].

### 3.2. Laparoscopic/Open Surgical Approach

The use of a laparoscopic or open approach allows repeated placement of RFA electrodes at multiple sites to ablate larger tumors [59–66] (Table 4). Moreover, a hand-assisted technique can be applied safely and effectively to laparoscopic liver surgery and offers the advantages of intraoperative US, which provides better resolution of the number and location of liver tumors. The postoperative recovery of patients was shorter compared with that after an open surgical approach. Ishiko et al. reported that the surgical procedures consisted of 5 RFA sessions for tumors in the caudate lobe with hand-assisted laparoscopic surgery (HALS) and a postoperative CT scan demonstrated sufficient ablation in all patients and there was no surgical mortality [63]. The HALS approach has several advantages; it facilitates and expedites the procedure, reduces the stress factor on the surgeon, greatly improves exposure, and facilitates immediate and efficient control of bleeding vessels with the internal hand. However, the local treatment failure rate of the laparoscopic approach was higher in patients with HCC nodules situated deep within the liver and measuring 4 cm or more in diameter [81]. Great difficulty can be encountered during treatment of lesions in contact with the diaphragm. 

Although more invasive, open RFA can be performed more easily, and the puncture course of RF needle can be more widely selected than that during laparoscopic approach. Some have reported that patients undergoing radical open RFA demonstrated few ablation site recurrences even though the nodules measured more than 4 cm in diameter and/or there were more than three nodules [59, 62, 65].

### 3.3. Complications

A recent review indicated that complication rates for percutaneous, laparoscopic, and open RFA of hepatic tumors in 3670 patients were 7.2%, 9.5%, and 9.9%, respectively [82]. Overall, the frequency of major complications of percutaneous RFA ranged from 0.6%–8.9%, which was higher than that of PEI, but generally less than that of MCT [43]. Complications of percutaneous RFA reported in 2320 patients treated at 41 different hospitals in Italy indicate that the mortality rate was 0.3% with an overall complication rate of 7.1% [83, 84]. The authors described major complications (2.4% incidence) including death, hemorrhage, RFA needle-track seeding, RFA lesion abscess, perforation of gastrointestinal viscus, liver failure, biloma, biliary stricture, portal vein thrombosis, and hemothorax or pneumothorax requiring drainage, and minor complications (4.7% incidence) including pain, fever, and asymptomatic pleural effusion. Although Llovet et al. [85] reported that dissemination along puncture route was observed in 12.5% of their patients, dissemination might not occur at such a high frequency. This complication was almost absent in many reports from Japan [43]. 

Theoretically, a tumor that is contiguous to a large vessel is more likely to have some viable tumor cells following local thermal therapy because there is a significant tissue cooling effect caused by blood circulation of normal body temperature. Thus, the effort to thoroughly ablate the lesion with a safety margin under such conditions increases the total number of electrode insertions, and this may increase the risk of complications. Some investigators have suggested that tumor location is closely related to the risk of major complications. Central tumors close to the hepatic hilum were reported to be unsuitable for percutaneous RFA because of the risk of injuring adjacent bile ducts [7]. Moreover, peripheral tumors adjacent to extrahepatic organs were also suggested to be unsuitable because of the risk of heat injuries, such as intestinal perforation and pleural effusion [84, 86]. However, Teratani et al. reported that there was no difference in early complication rates according to tumor location [87]. The effort to achieve thorough ablation increased the total number of electrode insertions, and this may have led to an increase in complications. 

Not only elevating the survival rate and reducing the incidence of local recurrence but also avoiding complications as much as possible are major tasks. To minimize complications of RFA, knowledge of risk factors and prevention methods is required. In addition, because early and accurate diagnosis is necessary for the appropriate management of complications, physicians should be familiar with all features of complication.

## 4. Future Perspective

Currently, a multicenter randomized controlled study (prospective randomized study of surgery or RFA for early HCC: SURF Trial) is underway in Japan, involving patients with 3 or fewer tumors 3 cm or smaller for which both hepatectomy and RFA are applicable [88], and a large global study is currently underway (the Sorafenib as Adjuvant Treatment in the Prevention of Recurrence of Hepatocellular Carcinoma (STORM) trial), looking at the efficacy of sorafenib therapy after potentially curative treatment with liver resection or RFA.

## 5. Conclusion

Here, we have assessed the role of RFA in the overall therapeutic strategy for patients with HCC and highlighted deficiencies in current knowledge. We intend to strive for a balanced discussion between the tendency to overemphasize the potential advantages of RFA and the tendency to understate a potentially useful treatment. Percutaneous RFA can achieve the same overall and disease-free survival rates as surgical resection for patients with small HCC, while causing few side effects. Percutaneous RFA combined with TACE will make the treatment of larger tumors a clinically viable treatment alternative. The use of a laparoscopic or open approach allows repeated placement of RFA electrodes at multiple sites to ablate larger tumors. In addition, an accurate evaluation of treatment response is very important to secure successful RFA therapy since a sufficient safety margin (at least 0.5 cm) can prevent local tumor recurrence. Adjuvant therapy, such as molecular targeted therapies following curative therapy, is expected to further improve survival after RFA.

##  Authors' Contributions

Y. Minami drafted the paper and wrote the final version of the paper. M. Kudo reviewed and approved the final version of the paper.

## Figures and Tables

**Table 1:** Survivals: RFA versus hepatic resection for HCC.

Author/Year	Study type	*n* (RFA/resection)	Mean tumor size (cm) (RFA/resection)	Overall survival (%) (RFA versus resection)	*P*
Chen et al. [15], 2006	RCT	90/90	ND/ND	65.9 versus 64.0 (4-year)	NS
Huang et al. [16], 2010	RCT	115/115	ND/ND	54.78 versus 75.65 (5-year)	.001
Vivarelli et al. [17], 2004	Retrospective	79/79	ND/ND	33 versus 65 (3-year)	.002
Montorsi et al. [18], 2005	Prospective	58/40	ND/ND	30 versus 53 (4-year)	.018
Ogihara et al. [19], 2005	Retrospective	40/47	4.6/7.4	39 versus 31 (5-year)	.79
Wakai et al. [20], 2006	Retrospective	64/85	ND/ND	30 versus 53 (10-year)	.012
Guglielmi et al. [21], 2008	Retrospective	23/33	ND/ND	45 versus 55 (5-year)	.7
Abu-Hilal et al. [22], 2008	Retrospective	34/34	3.0/3.8	57 versus 56 (5-year)	.3
Hiraoka et al. [23], 2008	Retrospective	105/59	ND/ND	59.3 versus 59.4 (5-year)	NS
Ueno et al. [24], 2009	Retrospective	123/110	2.0/2.7	63 versus 80 (5-year)	.06
Takayama et al. [25], 2009	Retrospective	1315/1235	1.6/1.8	95 versus 94 (2-year)	.28

HCC: hepatocellular carcinoma; ND: not described; NS: not significant; RFA: radiofrequency ablation.

**Table 2:** Local tumor progression rates after RFA for HCC.

Author	Year	*n*	Tumor size (mean, cm)	Follow-up period (mean, months)	Local tumor progression rate (%)
Rossi et al. [26]	1996	41	2.3	22.6	5.0
Buscarimi et al. [27]	2001	60	ND	26.8	14
Choi et al. [28]	2004	53	2.1	23	21
Lu et al. [29]	2005	87	2.5	12.7	5.8
Shiina et al. [30]	2005	118	ND	34.8	1.7
Solmi et al. [31]	2006	63	2.8	32.3	41
Hänsler et al. [32]	2007	21	4.2	ND	21
Waki et al. [33]	2010	88	ND	36	4.8
Li et al. [34]	2010	117	2.4	21	9.4

HCC: hepatocellular carcinoma; ND: not described; RFA: radiofrequency ablation.

**Table 3:** Survivals: RFA combined with TACE versus RFA alone for HCC.

Author/year	*n* (TACE+RFA/RFA)	Tumor size (mean, cm) (TACE+RFA/RFA)	Overall survival (%) (TACE+RFA/RFA)	*P*
Kitamoto et al. [49]/2003	10/16	3.9/3.4	ND	
Wang et al. [50]/2007	43/40	ND	68.3/57.6 (1-year)	<.05
Shibata et al. [51]/2009	46/43	ND	84.8/84.5 (3-year)	.515
Morimoto et al. [52]/2010	19/18	3.6/3.7	93/80 (3-year)	.369
Peng et al. [47]/2010	120/120	ND	50/42 (5-year)	.045

HCC: hepatocellular carcinoma; ND: not described; RFA: radiofrequency ablation, TACE: trans catheter arterial chemoembolization.

**Table 4:** Laparoscopic/open RFA for liver malignancies: local tumor progressions and survivals.

Author/year	Arms	*n*	Tumor size (mean, cm)	Follow-up period (mean, months)	Local tumor progression	Survival (%)
Topal et al. [59]/2003	LS/open	9/9	3.8/3.5	12.2	1/9, 0/9	ND
Berber et al. [60]/2005	LS	66	4.1	25.3	ND	38% (3-year)
Hildebrand et al. [61]/2007	LS	14	ND	23.2	1/14	ND
Minami et al. [62]/2007	open	30	3.2	18.9	1/30	71.6% (3-year)
Ishiko et al. [63]/2008	HALS	5	ND	32.2	1/5	ND
Ballem et al. [64]/2008	LS	104	3.5	23	ND	21% (3-year)
Tanaka et al. [65]/2009	open	26	ND	ND	1/26	ND
Salama et al. [66]/2010	LS	72	ND	14.3	2/72	ND

HALS: hand-assisted laparoscopic surgery; LS: laparoscopy; ND: not described; RFA: radiofrequency ablation.

## References

[1] Cho YK, Kim JK, Kim WT, Chung JW (2010). Hepatic resection versus radiofrequency ablation for very early stage hepatocellular carcinoma: a Markov model analysis. *Hepatology*.

[2] Rust C, Gores GJ (2001). Locoregional management of hepatocellular carcinoma. Surgical and ablation therapies. *Clinics in Liver Disease*.

[3] Lee WS, Yun SH, Chun HK (2008). Clinical outcomes of hepatic resection and radiofrequency ablation in patients with solitary colorectal liver metastasis. *Journal of Clinical Gastroenterology*.

[4] Mulier S, Ruers T, Jamart J, Michel L, Marchal G, Ni Y (2008). Radiofrequency ablation versus resection for resectable colorectal liver metastases: time for a randomized trial? An update. *Digestive Surgery*.

[5] Bartolozzi C, Lencioni R (1996). Ethanol injection for the treatment of hepatic tumours. *European Radiology*.

[6] Okada S (1999). Local ablation therapy for hepatocellular carcinoma. *Seminars in Liver Disease*.

[7] McGahan JP, Dodd GD (2001). Radiofrequency ablation of the liver: current status. *American Journal of Roentgenology*.

[8] Shiina S, Teratani T, Obi S, Hamamura K, Koike Y, Omata M (2002). Nonsurgical treatment of hepatocellular carcinoma: from percutaneous ethanol injection therapy and percutaneous microwave coagulation therapy to radiofrequency ablation. *Oncology*.

[9] Gillams AR (2003). Radiofrequency ablation in the management of liver tumours. *European Journal of Surgical Oncology*.

[10] Minami Y, Kudo M (2010). Radiofrequency ablation of hepatocellular carcinoma: current status. *World Journal of Radiology*.

[11] McGahan JP, Brock JM, Tesluk H, Gu WZ, Schneider P, Browning PD (1992). Hepatic ablation with use of radio-frequency electrocautery in the animal model. *Journal of Vascular and Interventional Radiology *.

[12] McGahan JP, Browning PD, Brock JM, Tesluk H (1990). Hepatic ablation using radiofrequency electrocautery. *Investigative Radiology*.

[13] Goldberg SN, Gazelle GS, Halpern EF, Rittman WJ, Mueller PR, Rosenthal DI (1996). Radiofrequency tissue ablation: importance of local temperature along the electrode tip exposure in determining lesion shape and size. *Academic Radiology*.

[14] The Japan Society of Hepatology (2007). *Consensus-Based Clinical Practice Manual*.

[15] Chen MS, Li JQ, Zheng Y (2006). A prospective randomized trial comparing percutaneous local ablative therapy and partial hepatectomy for small hepatocellular carcinoma. *Annals of Surgery*.

[16] Huang J, Yan L, Cheng Z (2010). A randomized trial comparing radiofrequency ablation and surgical resection for HCC conforming to the Milan criteria. *Annals of Surgery*.

[17] Vivarelli M, Guglielmi A, Ruzzenente A (2004). Surgical resection versus percutaneous radiofrequency ablation in the treatment of hepatocellular carcinoma on cirrhotic liver. *Annals of Surgery*.

[18] Montorsi M, Santambrogio R, Bianchi P (2005). Survival and recurrences after hepatic resection or radiofrequency for hepatocellular carcinoma in cirrhotic patients: a multivariate analysis. *Journal of Gastrointestinal Surgery*.

[19] Ogihara M, Wong LL, Machi J (2005). Radiofrequency ablation versus surgical resection for single nodule hepatocellular carcinoma: long-term outcomes. *HPB*.

[20] Wakai T, Shirai Y, Suda T (2006). Long-term outcomes of hepatectomy vs percutaneous ablation for treatment of hepatocellular carcinoma ≤ 4 cm. *World Journal of Gastroenterology*.

[21] Guglielmi A, Ruzzenente A, Valdegamberi A (2008). Radiofrequency ablation versus surgical resection for the treatment of hepatocellular carcinoma in cirrhosis. *Journal of Gastrointestinal Surgery*.

[22] Abu-Hilal M, Primrose JN, Casaril A, McPhail MJW, Pearce NW, Nicoli N (2008). Surgical resection versus radiofrequency ablation in the treatment of small unifocal hepatocellular carcinoma. *Journal of Gastrointestinal Surgery*.

[23] Hiraoka A, Horiike N, Yamashita Y (2008). Efficacy of radiofrequency ablation therapy compared to surgical resection in 164 patients in Japan with single hepatocellular carcinoma smaller than 3 cm, along with report of complications. *Hepato-Gastroenterology*.

[24] Ueno S, Sakoda M, Kubo F (2009). Surgical resection versus radiofrequency ablation for small hepatocellular carcinomas within the Milan criteria. *Journal of Hepato-Biliary-Pancreatic Surgery*.

[25] Takayama T, Makuuchi M, Hasegawa K (2010). Single HCC smaller than 2 cm: surgery or ablation?: surgeon’s perspective. *Journal of Hepato-Biliary-Pancreatic Sciences*.

[26] Rossi S, Di Stasi M, Buscarini E (1996). Percutaneous RF interstitial thermal ablation in the treatment of hepatic cancer. *American Journal of Roentgenology*.

[27] Buscarini L, Buscarini E, Di Stasi M, Vallisa D, Quaretti P, Rocca A (2001). Percutaneous radiofrequency ablation of small hepatocellular carcinoma: long-term results. *European Radiology*.

[28] Choi D, Lim HK, Kim MJ (2004). Recurrent hepatocellular carcinoma: percutaneous radiofrequency ablation after hepatectomy. *Radiology*.

[29] Lu DSK, Yu NC, Raman SS (2005). Percutaneous radiofrequency ablation of hepatocellular carcinoma as a bridge to liver transplantation. *Hepatology*.

[30] Shiina S, Teratani T, Obi S (2005). A randomized controlled trial of radiofrequency ablation with ethanol injection for small hepatocellular carcinoma. *Gastroenterology*.

[31] Solmi L, Nigro G, Roda E (2006). Therapeutic effectiveness of echo-guided percutaneous radiofrequency ablation therapy with a LeVeen needle electrode in hepatocellular carcinoma. *World Journal of Gastroenterology*.

[32] Hänsler J, Frieser M, Tietz V (2007). Percutaneous radiofrequency ablation of liver tumors using multiple saline-perfused electrodes. *Journal of Vascular and Interventional Radiology*.

[33] Waki K, Aikata H, Katamura Y (2010). Percutaneous radiofrequency ablation as first-line treatment for small hepatocellular carcinoma: results and prognostic factors on long-term follow up. *Journal of Gastroenterology and Hepatology*.

[34] Li W-H, Ma K-W, Cheng M (2010). Radiofrequency ablation for hepatocellular carcinoma: a survival analysis of 117 patients. *ANZ Journal of Surgery*.

[35] Kudo M (2010). Radiofrequency ablation for hepatocellular carcinoma: updated review in 2010. *Oncology*.

[36] Kudo M, Okanoue T (2007). Management of hepatocellular carcinoma in Japan: consensus-based clinical practice manual proposed by the Japan Society of Hepatology. *Oncology*.

[37] Bruix J, Sherman M (2005). Management of hepatocellular carcinoma. *Hepatology*.

[38] Ni Y, Mulier S, Miao Y, Michel L, Marchal G (2005). A review of the general aspects of radiofrequency ablation. *Abdominal Imaging*.

[39] Ni Y, Chen F, Mulier S (2006). Magnetic resonance imaging after radiofrequency ablation in a rodent model of liver tumor: tissue characterization using a novel necrosis-avid contrast agent. *European Radiology*.

[40] Mori K, Fukuda K, Asaoka H (2009). Radiofrequency ablation of the liver: determination of ablative margin at MR imaging with impaired clearance of ferucarbotran—feasibility study. *Radiology*.

[41] Kim YS, Rhim H, Cho OK, Koh BH, Kim Y (2006). Intrahepatic recurrence after percutaneous radiofrequency ablation of hepatocellular carcinoma: analysis of the pattern and risk factors. *European Journal of Radiology*.

[42] Lim HK, Choi D, Lee WJ (2001). Hepatocellular carcinoma treated with percutaneous radio-frequency ablation: evaluation with follow-up multiphase helical CT. *Radiology*.

[43] Kudo M (2004). Local ablation therapy for hepatocellular carcinoma: current status and future perspectives. *Journal of Gastroenterology*.

[44] Takahashi S, Kudo M, Chung H (2007). Initial treatment response is essential to improve survival in patients with hepatocellular carcinoma who underwent curative radiofrequency ablation therapy. *Oncology*.

[45] Lau WY, Lai ECH (2008). Hepatocellular carcinoma: current management and recent advances. *Hepatobiliary and Pancreatic Diseases International*.

[46] Arii S, Sata M, Sakamoto M (2010). Management of hepatocellular carcinoma: report of consensus meeting in the 45th annual meeting of the Japan Society of Hepatology (2009). *Hepatology Research*.

[47] Peng ZW, Chen MS, Liang HH (2010). A case-control study comparing percutaneous radiofrequency ablation alone or combined with transcatheter arterial chemoembolization for hepatocellular carcinoma. *European Journal of Surgical Oncology*.

[48] Yamakado K, Nakatsuka A, Takaki H (2008). Early-stage hepatocellular carcinoma: radiofrequency ablation combined with chemoembolization versus hepatectomy. *Radiology*.

[49] Kitamoto M, Imagawa M, Yamada H (2003). Radiofrequency ablation in the treatment of small hepatocellular carcinomas: comparison of the radiofrequency effect with and without chemoembolization. *American Journal of Roentgenology*.

[50] Wang YB, Chen MH, Yan K, Yang W, Dai Y, Yin SS (2007). Quality of life after radiofrequency ablation combined with transcatheter arterial chemoembolization for hepatocellular carcinoma: comparison with transcatheter arterial chemoembolization alone. *Quality of Life Research*.

[51] Shibata T, Isoda H, Hirokawa Y, Arizono S, Shimada K, Togashi K (2009). Small hepatocellular carcinoma: is radiofrequency ablation combined with transcatheter arterial chemoembolization more effective than radiofrequency ablation alone for treatment?. *Radiology*.

[52] Morimoto M, Numata K, Kondou M, Nozaki A, Morita S, Tanaka K (2010). Midterm outcomes in patients with intermediate-sized hepatocellular carcinoma: a randomized controlled trial for determining the efficacy of radiofrequency ablation combined with transcatheter arterial chemoembolization. *Cancer*.

[53] Uehara T, Hirooka M, Ishida K (2007). Percutaneous ultrasound-guided radiofrequency ablation of hepatocellular carcinoma with artificially induced pleural effusion and ascites. *Journal of Gastroenterology*.

[54] Koda M, Ueki M, Maeda Y (2004). Percutaneous sonographically guided radiofrequency ablation with artificial pleural effusion for hepatocellular carcinoma located under the diaphragm. *American Journal of Roentgenology*.

[55] Minami Y, Kudo M, Kawasaki T, Chung H, Ogawa C, Shiozaki H (2004). Percutaneous radiofrequency ablation guided by contrast-enhanced harmonic sonography with artificial pleural effusion for hepatocellular carcinoma in the hepatic dome. *American Journal of Roentgenology*.

[56] Minami Y, Kudo M, Kawasaki T (2003). Percutaneous ultrasound-guided radiofrequency ablation with artificial pleural effusion for hepatocellular carcinoma in the hepatic dome. *Journal of Gastroenterology*.

[57] Rhim H, Lim HK, Kim YS, Choi D (2008). Percutaneous radiofrequency ablation with artificial ascites for hepatocellular carcinoma in the hepatic dome: initial experience. *American Journal of Roentgenology*.

[58] Song I, Rhim H, Lim HK, Kim YS, Choi D (2009). Percutaneous radiofrequency ablation of hepatocellular carcinoma abutting the diaphragm and gastrointestinal tracts with the use of artificial ascites: safety and technical efficacy in 143 patients. *European Radiology*.

[59] Topal B, Aerts R, Penninckx F (2003). Laparoscopic radiofrequency ablation of unresectable liver malignancies: feasibility and clinical outcome. *Surgical Laparoscopy, Endoscopy and Percutaneous Techniques*.

[60] Berber E, Rogers S, Siperstein A (2005). Predictors of survival after laparoscopic radiofrequency thermal ablation of hepatocellular cancer: a prospective study. *Surgical Endoscopy and Other Interventional Techniques*.

[61] Hildebrand P, Kleemann M, Roblick U, Mirow L, Birth M, Bruch HP (2007). Laparoscopic radiofrequency ablation of unresectable hepatic malignancies: indication, limitation and results. *Hepato-Gastroenterology*.

[62] Minami Y, Kawasaki T, Kudo M (2007). Treatment of large and/or multiple hepatic malignancies: open surgical approaches of radiofrequency ablation. *Hepato-Gastroenterology*.

[63] Ishiko T, Beppu T, Sugiyama S (2008). Radiofrequency ablation with hand-assisted laparoscopic surgery for the treatment of hepatocellular carcinoma in the caudate lobe. *Surgical Laparoscopy, Endoscopy and Percutaneous Techniques*.

[64] Ballem N, Berber E, Pitt T, Siperstein A (2008). Laparoscopic radiofrequency ablation of unresectable hepatocellular carcinoma: long-term follow-up. *HPB*.

[65] Tanaka S, Shimada M, Shirabe K (2009). Surgical radiofrequency ablation for treatment of hepatocellular carcinoma: an endoscopic or open approach. *Hepato-Gastroenterology*.

[66] Salama IA, Korayem E, Elabd O, El-Refaie A (2010). Laparoscopic ultrasound with radiofrequency ablation of hepatic tumors in cirrhotic patients. *Journal of Laparoendoscopic and Advanced Surgical Techniques*.

[67] Takayasu K, Muramatsu Y, Asai S, Muramatsu Y, Kobayashi T (1999). CT fluoroscopy-assisted needle puncture and ethanol injection for hepatocellular carcinoma: a preliminary study. *American Journal of Roentgenology*.

[68] Ding H, Kudo M, Onda H, Suetomi Y, Minami Y, Maekawa K (2001). Hepatocellular carcinoma: depiction of tumor parenchymal flow with intermittent harmonic power Doppler US during the early arterial phase in dual-display mode. *Radiology*.

[69] Kudo M (2001). Contrast harmonic ultrasound is a breakthrough technology in the diagnosis and treatment of hepatocellular carcinoma. *Journal of Medical Ultrasonics*.

[70] Ding H, Kudo M, Onda H (2001). Evaluation of posttreatment response of hepatocellular carcinoma with contrast-enhanced coded phase-inversion harmonic US: comparison with dynamic CT. *Radiology*.

[71] Quaia E, Calliada F, Bertolotto M (2004). Characterization of focal liver lesions with contrast-specific US modes and a sulfur hexafluoride-filled microbubble contrast agent: diagnostic performance and confidence. *Radiology*.

[72] Wang Z, Tang J, An L (2007). Contrast-enhanced ultrasonography for assessment of tumor vascularity in hepatocellular carcinoma. *Journal of Ultrasound in Medicine*.

[73] Leen E, Angerson WJ, Yarmenitis S (2002). Multi-centre clinical study evaluating the efficacy of SonoVue*™* (BR1), a new ultrasound contrast agent in Doppler investigation of focal hepatic lesions. *European Journal of Radiology*.

[74] Kudo M, Minami Y (2003). Radiofrequency ablation therapy under harmonic imaging guidance for the recurring cancer after local therapy for HCC: a randomized controlled study with RFA under B-mode guidance. *Ultrasound in Medicine and Biology*.

[75] Minami Y, Kudo M, Kawasaki T, Chung H, Ogawa C, Shiozaki H (2004). Treatment of hepatocellular carcinoma with percutaneous radiofrequency ablation: usefulness of contrast harmonic sonography for lesions poorly defined with B-mode sonography. *American Journal of Roentgenology*.

[76] Minami Y, Kudo M, Chung H (2007). Contrast harmonic sonography-guided radiofrequency ablation therapy versus B-mode sonography in hepatocellular carcinoma: prospective randomized controlled trial. *American Journal of Roentgenology*.

[77] Minami Y, Kudo M (2009). Contrast-enhanced harmonic ultrasound imaging in ablation therapy for primary hepatocellular carcinoma. *World Journal of Radiology*.

[78] Minami Y, Kudo M, Hatanaka K (2010). Radiofrequency ablation guided by contrast harmonic sonography using perfluorocarbon microbubbles (Sonazoid) for hepatic malignancies: an initial experience. *Liver International*.

[79] Minami Y, Kudo M, Chung H (2007). Percutaneous radiofrequency ablation of sonographically unidentifiable liver tumors: feasibility and usefulness of a novel guiding technique with an integrated system of computed tomography and sonographic images. *Oncology*.

[80] Minami Y, Chung H, Kudo M (2008). Radiofrequency ablation of hepatocellular carcinoma: value of virtual CT sonography with magnetic navigation. *American Journal of Roentgenology*.

[81] Santambrogio R, Opocher E, Montorsi M (2008). Laparoscopic radiofrequency ablation of hepatocellular carcinoma: a critical review from the surgeon’s perspective. *Journal of Ultrasound*.

[82] Mulier S, Mulier P, Ni Y (2002). Complications of radiofrequency coagulation of liver tumours. *British Journal of Surgery*.

[83] Bouza C, López-Cuadrado T, Alcázar R, Saz-Parkinson Z, Amate JM (2009). Meta-analysis of percutaneous radiofrequency ablation versus ethanol injection in hepatocellular carcinoma. *BMC Gastroenterology*.

[84] Livraghi T, Solbiati L, Meloni MF, Gazelle GS, Halpern EF, Goldberg SN (2003). Treatment of focal liver tumors with percutaneous radio-frequency ablation: complications encountered in a multicenter study. *Radiology*.

[85] Llovet JM, Vilana R, Brú C (2001). Increased risk of tumor seeding after percutaneous radiofrequency ablation for single hepatocellular carcinoma. *Hepatology*.

[86] Meloni MF, Goldberg SN, Moser V, Piazza G, Livraghi T (2002). Colonic perforation and abscess following radiofrequency ablation treatment of hepatoma. *European Journal of Ultrasound*.

[87] Teratani T, Yoshida H, Shiina S (2006). Radiofrequency ablation for hepatocellular carcinoma in so-called high-risk locations. *Hepatology*.

[88] Hasegawa K, Kokudo N, Shiina S, Tateishi R, Makuuchi M (2010). Surgery versus radiofrequency ablation for small hepatocellular carcinoma: start of a randomized controlled trial (SURF trial). *Hepatology Research*.

